# Performance Evaluation of the Becton Dickinson FACSPresto^™^ Near-Patient CD4 Instrument in a Laboratory and Typical Field Clinic Setting in South Africa

**DOI:** 10.1371/journal.pone.0156266

**Published:** 2016-05-25

**Authors:** Lindi-Marie Coetzee, Keshendree Moodley, Deborah Kim Glencross

**Affiliations:** 1 National Health Laboratory Service of South Africa (NHLS), Charlotte Maxeke Hospital, CD4 Laboratory, Parktown, Johannesburg, South Africa; 2 Department of Molecular Medicine and Haematology, Faculty of Health Sciences, University of the Witwatersrand, 7 York Road, Parktown, 2198, Johannesburg, South Africa; Ghent University, BELGIUM

## Abstract

**Background:**

The BD-FACSPresto^™^ CD4 is a new, point-of-care (POC) instrument utilising finger-stick capillary blood sampling. This study evaluated its performance against predicate CD4 testing in South Africa.

**Methods:**

Phase-I testing: HIV+ patient samples (n = 214) were analysed on the Presto^™^ under ideal laboratory conditions using venous blood. During Phase-II, 135 patients were capillary-bled for CD4 testing on FACSPresto^™^, performed according to manufacturer instruction. Comparative statistical analyses against predicate PLG/CD4 method and industry standards were done using GraphPad Prism 6. It included Bland-Altman with 95% limits of agreement (LOA) and percentage similarity with coefficient of variation (%CV) analyses for absolute CD4 count (cells/μl) and CD4 percentage of lymphocytes (CD4%).

**Results:**

In Phase-I, 179/217 samples yielded reportable results with Presto^™^ using venous blood filled cartridges. Compared to predicate, a mean bias of 40.4±45.8 (LOA of -49.2 to 130.2) and %similarity (%CV) of 106.1%±7.75 (7.3%) was noted for CD4 absolute counts. In Phase-2 field study, 118/135 capillary-bled Presto^™^ samples resulted CD4 parameters. Compared to predicate, a mean bias of 50.2±92.8 (LOA of -131.7 to 232) with %similarity (%CV) 105%±10.8 (10.3%), and 2.87±2.7 (LOA of -8.2 to 2.5) with similarity of 94.7±6.5% (6.83%) noted for absolute CD4 and CD4% respectively. No significant clinical differences were indicated for either parameter using two sampling methods.

**Conclusion:**

The Presto^™^ produced remarkable precision to predicate methods, irrespective of venous or capillary blood sampling. A consistent, clinically insignificant over-estimation (5–7%) of counts against PLG/CD4 and equivalency to FACSCount^™^ was noted. Further field studies are awaited to confirm longer-term use.

## Introduction

CD4 lymphocyte enumeration has long been used for staging of HIV and identifying HIV patients eligible for-or monitoring patients already on anti-retroviral therapy (ART) [1–4]. Although thresholds for treatment initiation have changed over time (i.e. <250cells/μl, <350cells/μl and <500cells/μl). [5–8]. CD4 is still considered essential to establish disease progression as well as for early detection of opportunistic co-infections such as *Cryptococcus Neoformans* (CrAg) in patients with CD4<100cells/μl [7, 9, 10]. Point-of-care (POC) CD4 technologies are generally used to improve access to CD4 testing and expand enrollment of HIV+ patients onto treatment programs [11–17]. These include smaller flow cytometry systems like HumaCount^™^, CD4NOW (previously known as PointCareNOW) (Human Diagnostics Worldwide, Germany), Partec CyFlow^®^ miniPOC (Sysmex-Partec GmbH, Germany) and Millipore Muse^®^ (Merck Millipore, Darmstadt, Germany), while other instrument based systems introduced microfluidics technology to count cells on cartridges, like PIMA^™^ (Alere, Waltham, MA), Daktari^™^ (Daktari Diagnostics, MA, USA) and mBio (mBio Diagnostics, Boulder, CO). Simpler disposable lateral flow strip systems like Visitect (Omega Diagnostics) and Zyomyx MyT4 (Mylan, USA) offer instrument free options [18–21]. Several published papers describe validation of these systems with varying approaches, reference platforms and outcomes [22–26]. The PIMA point-of-care instrument has been commercially available the longest and several validation, implementation and costing studies have been reported, including a recent meta-analysis of POC technology [11, 26–33].

POC technologies are typically used by nursing and other clinic personnel, who may not necessarily be adequately trained in good clinical laboratory practice (GCLP). It is therefore important to evaluate existing and emerging POC CD4 systems for accuracy, reproducibility, sensitivity and specificity in the context in which they are prescribed for use. The aim of this study was to evaluate the performance of the newly launched Becton Dickinson FACSPresto^™^ (BD Biosciences, San Jose CA) near-patient Point-of-Care CD4 counter, both as the instrument is intended for use in the field, in a typical primary health care clinic where a point-of-care system would ideally be used as well as in a laboratory setting using venous blood under ideal laboratory conditions.

## Methods

### Purpose of validation

The evaluation of the FACSPresto^™^ Near-Patient CD4 instrument was done as part of NHLS internal requirements for evaluation of new technology through the Health Technology Assessment Unit (HTA) at the Charlotte Maxeke Johannesburg Academic Hospital (CMJAH) CD4 reference and research laboratory, in accordance with international guidelines [34]. Ethics approval was obtained through the University of the Witwatersrand (M121020 and M0909816). After initial equipment installation and training by the manufacturer, performance qualification (PQ) of the instrument was done in two phases, described below.

### Instrument Description

The FACSPresto^™^ Near-Patient CD4 analyser is a dedicated point-of-care system, using disposable cartridges with dried down reagents to detect absolute CD4 counts and CD4 percentage of lymphocytes. In addition, the cartridge also analyses the sample for absolute hemoglobin concentration (Hb) (not evaluated in this study). The cartridges can be filled with venous or capillary blood, with no additional sample preparation of quality control procedures necessary [35].

### Study design

#### Phase I

Phase I was done under ideal laboratory conditions to assess the baseline accuracy and precision of the instrument, compared to the predicate method of CD4 testing in the NHLS. The latter, i.e. FlowCare^™^ Panleuco-gated (PLG/CD4) 2-color (CD45 FITC/CD4-RD1) single platform cell enumeration (with addition of Flow Count fluorospheres, Beckman Coulter) [36–42]. Testing was done on a Beckman Coulter MPL/CellMek fully automated flow cytometer system using NHLS standardized standard operating procedures (SOPs). Remnant K3EDTA blood was obtained from the CD4 testing laboratory within 24 hours of predicate CD4 testing, and used to manually fill FACSPresto^™^ cartridges by pipetting EDTA blood into cartridges (n = 214). Up to ten patient samples (cartridges) were set up simultaneously and incubated at room temperature for 18 minutes before analysis on the instrument, with individual results printed within 2–3 minutes. A subset (n = 115/214) of samples were also tested on a 2nd BD platform (industry standard for small to medium volume CD4 testing), the BD FACSCount^™^ system [43–45], and the reference Tetrachrome^™^ (CD45FITC/CD4-RD1/CD8-ECD/CD3-PC5) T-cell enumeration method on the Beckman Coulter XL flow cytometer [46].

#### Phase II

Phase II of the study was done at the Witkoppen HIV counseling and testing (HCT) clinic in Johannesburg, where nursing staff (n = 2) filled FACSPresto^™^ cartridges with capillary blood from a finger-stick (using BD Vacutainer 1.5mm blade, 2mm depth for high blood flow), after patient consent, and as per manufacturer instructions. EDTA samples were taken for direct comparison of capillary vs. venous blood sampling including laboratory predicate CD4 analysis and manually EDTA-filled cartridges tested the laboratory-based FACSPresto^™^ instrument. In total, 135 samples were tested on site and sent for laboratory CD4 testing. For the purpose of this study, CD4 results obtained with the point-of-care instrument were not used for patient management.

### Quality Control

The FACSPresto^™^ instrument runs its own internal quality control checks at startup and each cartridge has built-in quality checks to verify reagents and sufficient sample volume. These run automatically at every cartridge analysis and are reported on the patient result printout. Additional accuracy and precision testing (reproducibility) was done as per internal validation requirements, with readily available reference stabilized-blood material, i.e. Immunotrol^™^ normal and low (Beckman Coulter) currently used for internal quality control of CD4 enumeration in NHLS CD4 laboratories. Each control was set up as 10 repeat tubes (the exercise was repeated on two consecutive days). Furthermore, 3 random patient samples were selected with representative CD4 counts of >500, ±500 and around 100 cells/μl. Ten replicates of each of the latter were prepared and analysed on the FACSPresto^™^ instrument and compared to the predicate PLG/CD4 results. As a final verification of accuracy and precision, a panel of retrospective external CD4 quality control samples from the last 10 trials (2014) of the NHLS CD4 Quality Assessment Scheme were analysed and a standard deviation index (SDI) value calculated for each result using the globally obtained trimmed mean and standard deviation values from the respective trial. The calculated SDI reflects the performance of the testing platform with expected outcome to be within 2 standard deviations of the trimmed pool mean for all participants, that includes different platforms andmethodologies [36, 46–48]. For the predicate methods (PLG/CD4 and Tetrachrome T-cell analysis), daily quality control was done in accordance to national SOP guidelines that includes daily assessment of laser alignment, accuracy and precision of result reporting and instrument stability [39].

### Statistical analyses

GraphPad Prism Software was used for statistical analyses and graphics. Where applicable, basic statistics were calculated to include minimum, maximum, mean and median values of parameters tested (CD4 absolute counts, CD4% of lymphocytes or Hemoglobin). %Similarity analyses were used to assess overall comparison between technologies or individual instruments [49]. The %similarity was calculated as (((Reference + Test)/2)/Reference)*100). For this calculation the %CV was used as an indication of precision to the predicate technology, with an acceptable %similarity CV of <10% between technologies. For the purpose of this study where both CD4 values of predicate and Presto were <100 cells/μl, % similarity calculation was corrected to 100% similar as these absolute counts <100cell/μl, would result in the same clinical decision. This correction was made, as the %similarity model “over-estimates” the difference (expressed as a percentage of the reference value) between samples if small values are compared. The overall comparison between PLG/CD4 and FACSPresto^™^ results was done using the non-parametric paired Wilcoxon-test to generate a p-value and calculate the significant difference between groups. Comparison of 2 or more data sets were analysed using repeated measures ANOVA with pairwise comparisons. Deming regression and Bland-Altman analyses were used to indicate overall agreement and bias between technologies for all data. Data was categorized according to current CD4 thresholds for therapy initiation in South Africa, which was 350 cells/μl (2013) and 500 cells/μl as per 2015 guidelines [6–8]. Categorized data was also used for both phases to assess the number of miss-classifications based on CD4 counts <100 (important for identifying samples for Cryptococcal antigen testing), <350 (previous WHO and South African HCT guidelines) or <500 (current South African HCT guidelines). For external quality assessment (EQA) results, an SDI value was calculated, using the global trimmed mean and Standard Deviation of all data submitted for a particular trial, as described previously (Lab mean minus Consensus group mean, divided by the Consensus group Standard Deviation).

## Results

### Phase 1 (Laboratory testing on venous samples)

A total of 214 EDTA random CD4 samples from adult patients were analysed using the PLG/CD4 predicate method. These samples were re-tested on the FACSPresto^™^ system within 24 hours using EDTA blood, manually pipetting into cartridges for analysis. Samples with a range of CD4 counts were included (1–1610 cells/μl; Table 1), with a mean CD4 value of 340cells/μl. Of the 214 samples analysed using the FACSPresto^™^ system, an absolute CD4 and CD4% of lymphocyte count was obtained in 179 of 215 samples (83%). The 35 samples that did not yield results were re-analysed and 31 did not return a result even on repeated analyses (overall failure rate of 16.4%). Four samples failed the internal cartridge quality control and yielded a result when repeated (regarded as true system errors accounting for <2% of tested samples). The majority of “no read” samples (n = 26/31, 84%) had a CD4 absolute count <100cells/μl, with 5 (16%) of ‘no-read’ errors noted where resulted counts were >100cells/μl. Although the manufacturer’s lower limit of detection is 50cells/μl, equal numbers of ‘no-read’ errors were seen in the category of samples with a count <50 and that of a CD4 counts between 50–99 cells/μl.

**Table 1:** Summary of Phase I validation descriptive statistics.

	Absolute CD4 count Range	PLG #CD4	Presto #CD4	PLG CD4%	Presto CD4%
**Number of values: n(%error rate)**	***<100***	54	**28 (48.2%)**	55	**28 (48.2%)**
	***100–349***	55	**52 (5.5%)**	54	**52 (5.5%)**
	***350–499***	53	**49 (7.5%)**	53	**49 (7.5%)**
	***>500***	52	**50 (3.8%)**	52	**50 (3.8%)**
	***>100***	160	**151 (5.6%)**	160	**151 (5.6%)**
	***All***	***214***	***179 (16*.*35%)***	***214***	***179 (16*.*35%)***
***Mean (Range)***	***All***	***340 (1–1610)***	***422 (6–1603)***	***17*.*1 (0*.*28–49*.*1)***	***20*.*16 (1*.*31–47*.*52)***
**%Similarity: Mean±STDev (%CV)**	***<100***	110.4±19.3 (17.5%)		103.5 ±14.33 (13.85%)	
	***100–349***	108.1 ±10.43 (9.65%)		106.3 ±10.59 (9.96%)	
(n = 50)	***350–499***	106.9 ± 5.61 (5.25%)		105.2 ±3.41 (3.24%)	
(n = 80)	***>500***	103.6 ± 4.04 (3.9%)		103.2 ± 2.96 (2.87%)	
(n = 129)	***<350***	108.9 ± 17.1 (10.75%)		105.3 ± 12.02 (11.41%)	
(n = 151)	***<500***	108.2 ±11.63 (10.75%)		105.3 ±9.67 "(9.19%)	
(n = 179)	***All***	***106*.*9 ± 10*.*3 (9*.*63%)***		***104*.*7 ± 8*.*39 (8*.*02%)***	
**Corrected for CD4<100 only**	***All (corrected)***	***106*.*1 ± 7*.*75 (7*.*31%)***		***104*.*5 ± 6*.*52 (6*.*24%)***	
**Bland-Altman: Bias ±STDev (95% LOA)**	***<100***	11.43 ± 17.29 (-22.46 to 45.32)		0.35 ± 1.5 (-2.7 to 3.45)	
	***100–349***	35.25 ± 39.59 (-42.3 to 112.8)		1.33 ± 1.82 (-2.24 to 4.91)	
	***350–499***	59.73 ± 48.53 (-35.4 to 154.9)		1.93 ± 1.14 (-0.3 to 4.16)	
(n = 50)	***>500***	43 ± 51.33 (-57.2 to 143.6)		1.57 ± 1.46 (-1.3 to 4.42)	
(n = 80)	***<350***	26.91 ± 35.28 (-42.23 to 96.1)		0.98 ± 1.79 (-2.53 to 4.51)	
(n = 129)	***<500***	39.38 ± 43.67 (-46.22 to 125.0)		1.34 ± 1.64 (-1.87 to 4.56)	
(n = 151)	***>100***	45.76 ± 47.46 (-47.25 to 138.8)		1.60 ± 1.52 (-1.37 to 4.58)	
(n = 179)	***All***	***40*.*39±45*.*82 (-49*.*42 to 130*.*2)***		***1*.*41 ± 1*.*6 (-1*.*7 to 4*.*5)***	
**%Relative Error ± STDev (95% CI of mean)**	ALL	13.8 ± 20.6 (10.74 to 16.82)		9.36 ± 16.8 (6.88 to 11.83)	
**Wilcoxon test p-value(p<0.05 significant)**	***<350***	P<0.0001		P<0.0001	
	***<500***	P<0.0001		P<0.0001	
	***All***	***P<0*.*0001***		***P<0*.*0001***	

Laboratory FACSPresto^**™**^ EDTA filled cartridges results compared to predicate method (MPL/CellMek PLG/CD4). %Similarity CV reflects precision of results generated on the Presto^™^ versus the predicate results. (S1 Table)

Direct comparison using the Wilcoxon test with post Mann-Whitney analysis showed differences between the predicate method (MPL/PLG CD4) and the manually EDTA filled cartridges tested on FACSPresto^™^ for both parameters tested (p<0.0001) (Table 1). %Similarity analysis confirmed a slight overall over-estimation by the Presto^™^ instrument of 7% for absolute CD4 count and 5% for CD4% of lymphocytes, while the coefficient of variance (CV) for the %similarity was <10%. No significant differences (106.1 to 106.9% for CD4 and 104.5 to 104.9% for CD4% with %CV<10%) in %similarity analyses was noted when all samples were compared to corrected for <100 cells/μl samples or analysis of samples with a CD4 count of >100cells/μl (excluding all <100 counts) (Table 1).

Bland-Altman analysis confirmed the over-estimation with a mean bias of 40.4±45.8 cells/μl (95% LOA of -49.4 to 130.2) for absolute CD4 counts and 1.4±1.6% (95% LOA of -1.7 to 4.5) for CD4% of lymphocytes (not clinically relevant). Re-analysis of data excluding samples with a CD4<100 changed the bias for absolute CD4 count to 45.7±47.7 and the bias for CD4% to 1.34±1.64 (not significant). The consistency in standard deviation of these observed biases confirmed the precision of the technology against the predicate method at all CD4 ranges tested. Sub-analysis of samples with a CD4 count >100<500 cells/μl showed that 19/108 (17%) patients would have been excluded for ART initiation at a treatment threshold cutoff of 500 cells/μl due to over-estimation of absolute CD4 counts by the FACSPresto^™^ instrument, while only one sample would have been misclassified for opportunistic cryptococcal antigen (CrAg) screening at count <100cells/μl (FACSPresto^™^ value of 95 vs. predicate of 150 cells/μl).

### Subset 4-way comparison (laboratory based)

**Fig 1:**
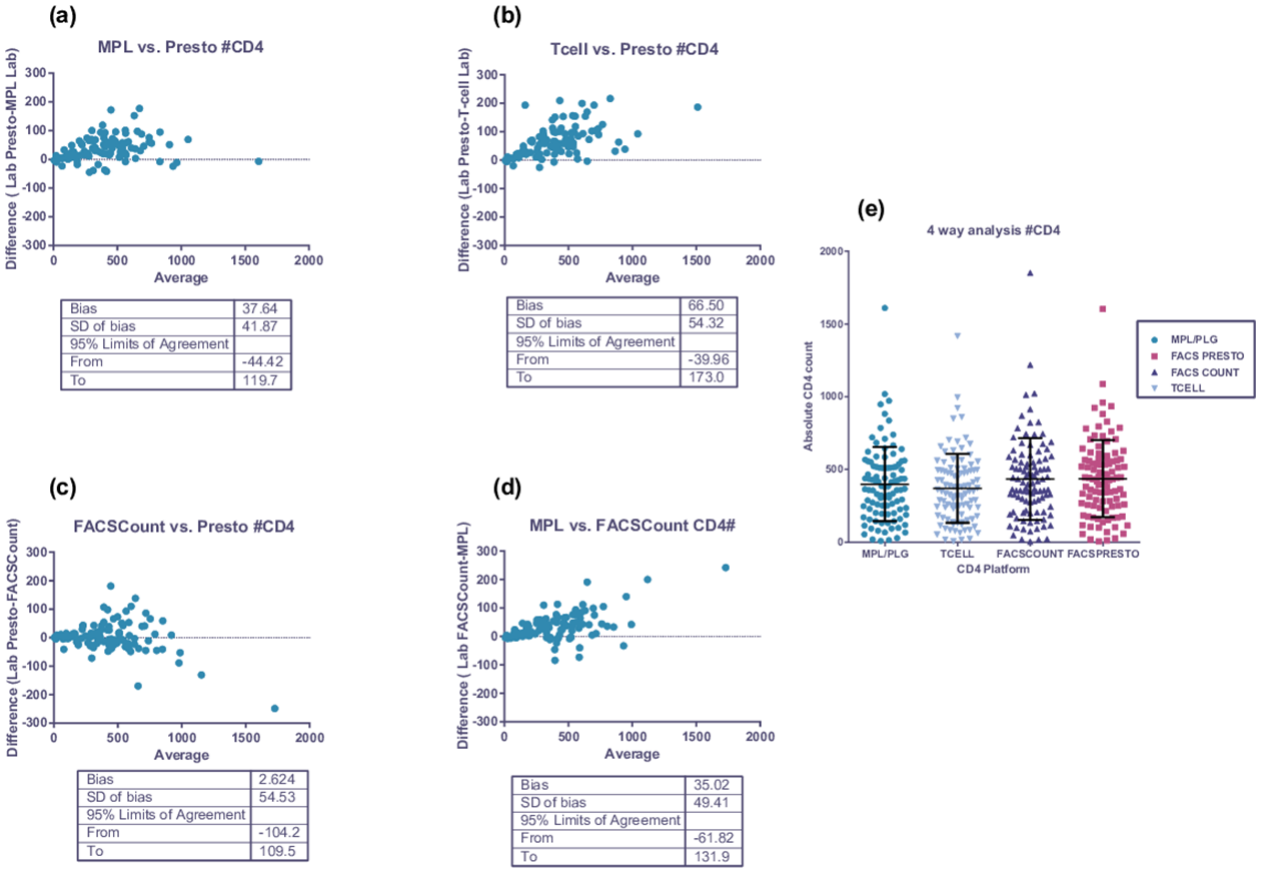
4 way sub-analysis of FACSPresto^™^ performance. Legend: This figure represents Bland-Altman analysis for absolute CD4 counts where results of the FACSPresto^™^ were compared to the following predicate methods: (a) MPL using PLG/CD4 platform; (b) T-cell tetrachrome and (c) BD FACSCount^™^. (d) Comparison of MPL (PLG/CD4) against FACSCount^™^ indicated equivalent performance to FACSPresto^™^ against this predicate. Comparative 4-way analysis of CD4 platform performance for absolute CD4 count enumeration (e) confirmed good correlation with no significant differences between datasets (p>0.05).

A subset of 115 patient samples were analysed in a 4-way comparison of FACSPresto^™^, FACSCount^™^, Tetrachrome^™^ 4-color T-cell method and the MPL PLG/CD4 predicate methods (Fig 1). Both Becton Dickinson instruments over-estimated absolute CD4 counts slightly relative to the predicate technology, confirmed with %similarity (105±5%) and Bland-Altman analysis (bias of 33.2±47.3 for FACSCount^™^ and 37.6±41.8 for FACSPresto^™^ against MPL/PLG predicate). Comparison of T-cell results vs. FACSPresto^™^ showed a slightly higher bias for absolute CD4 counts of 66.5±54.2 (%similarity of 108±7.2, CV 6.6%). Overall the %similarity CV values were <6% for absolute CD4 count comparisons. CD4% of lymphocytes 4-way comparison showed %similarity between 98–104% with correlating CV’s of <8%. These %CV values confirmed precision with the FACSPresto^™^ system against predicate methods. Bland-Altman analysis of CD4% of lymphocytes indicated a bias of -0.9±1.6 for FACSCount^™^ and 1.03±1.42 for FACSPresto^™^ against MPL PLG/CD4 respectively. The smallest biases were noted between instruments from the same manufacturer, i.e. Beckman Coulter MPL/CellMek PLG vs. XL T-cell (-27±42, 95% LOA -111 to 56 for absolute CD4 counts) or FACSCount^™^ and FACSPresto^™^ for both parameters (2.6±54, 95% LOA -104 to 110 for absolute CD4 count and 2.3±1,8, 95% LOA of -1.3 to 6 for CD4%) (p = 1, repeated measures ANOVA). Repeated measures ANOVA confirmed differences between platforms from different manufacturers (p<0.0001). (Fig 1e) (S1 Fig).

### Quality Control

**Fig 2:**
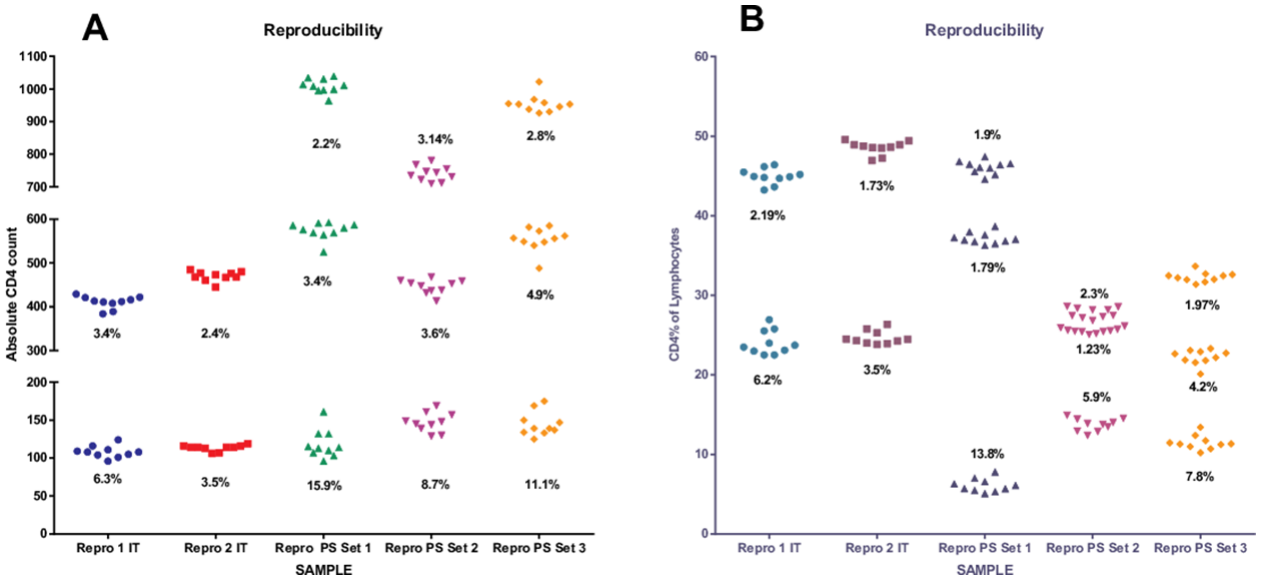
Reproducibility of FACSPresto^™^. Reproducibility of the FACSPresto^™^ system was assessed for (A) absolute CD4 counts and (B) CD4% of lymphocytes. Two sets of ten samples were analysed for Beckman Coulter Immunotrol (IT) normal and low (Repro 1 and 2 IT) and for nine patient samples (PS) with low, medium and high CD4 counts (3 per group), indicated as Repro PS Set 1–3. %CV results are indicated for each data set.

Beckman Coulter Immunotrol Normal and Low quality controls were used for reproducibility studies as this control material is readily available at our CD4 testing laboratories (2 sets of repros per control) (Fig 2) (S2 Fig). Overall, CV’s for absolute CD4 count and CD4% of lymphocytes was <8% (and <3% for the Hemoglobin results on FACSPresto^™^; data not shown). All results were within the package insert acceptable ranges for absolute CD4 counts and CD4% of lymphocytes. Ten replicates per sample was done on nine patient samples on the FACSPresto^™^ instruments (pipet filled cartridges) and %CV values confirmed tight reproducibility at medium and high absolute counts and CD4% values, with some instability in testing with 2 out of 3 samples with a CD4 count between 100–130 (CD4% 5–11%) having corresponding CV’s above 10% (Fig 2, patient sample set 1 and 3). %Similarity analysis between the FACSPresto^™^ and the package insert mean values indicated a slight under-estimating of absolute counts by 9% for normal and 4% for low controls, while an under-estimation of 2% were noted for CD4% of lymphocytes (both levels of controls). This related to a bias (Presto-Reference) on the normal Immunotrol absolute count (ref value 500cells/μl) of -59.7±32.9 (95% LOA -124 to 4.8) and -9.2±6.6 (95%LOA -22 to 3.6) for the low Immunotrol absolute count (ref value 120cells/μl). The corresponding CD4% of lymphocytes showed a bias of -0.55±2.1 (95% LOA -4.5 to 3.5) and -0.1±1.2 (95% LOA -2.5 to 2.3) for Immunotrol Normal and Low respectively (negligible).

In addition to the QC above, inter-instrument variability was assessed between two FACSPresto^™^ instruments in the laboratory on 20 random EDTA samples, using cartridge-filled technique. The %similarity for both absolute CD4 counts and CD4% of lymphocytes between instruments was 98 and 99% respectively with corresponding CV’s of <5% against the predicate, confirming equivalent instrument performance between FACSPresto^™^ instruments.

**Fig 3:**
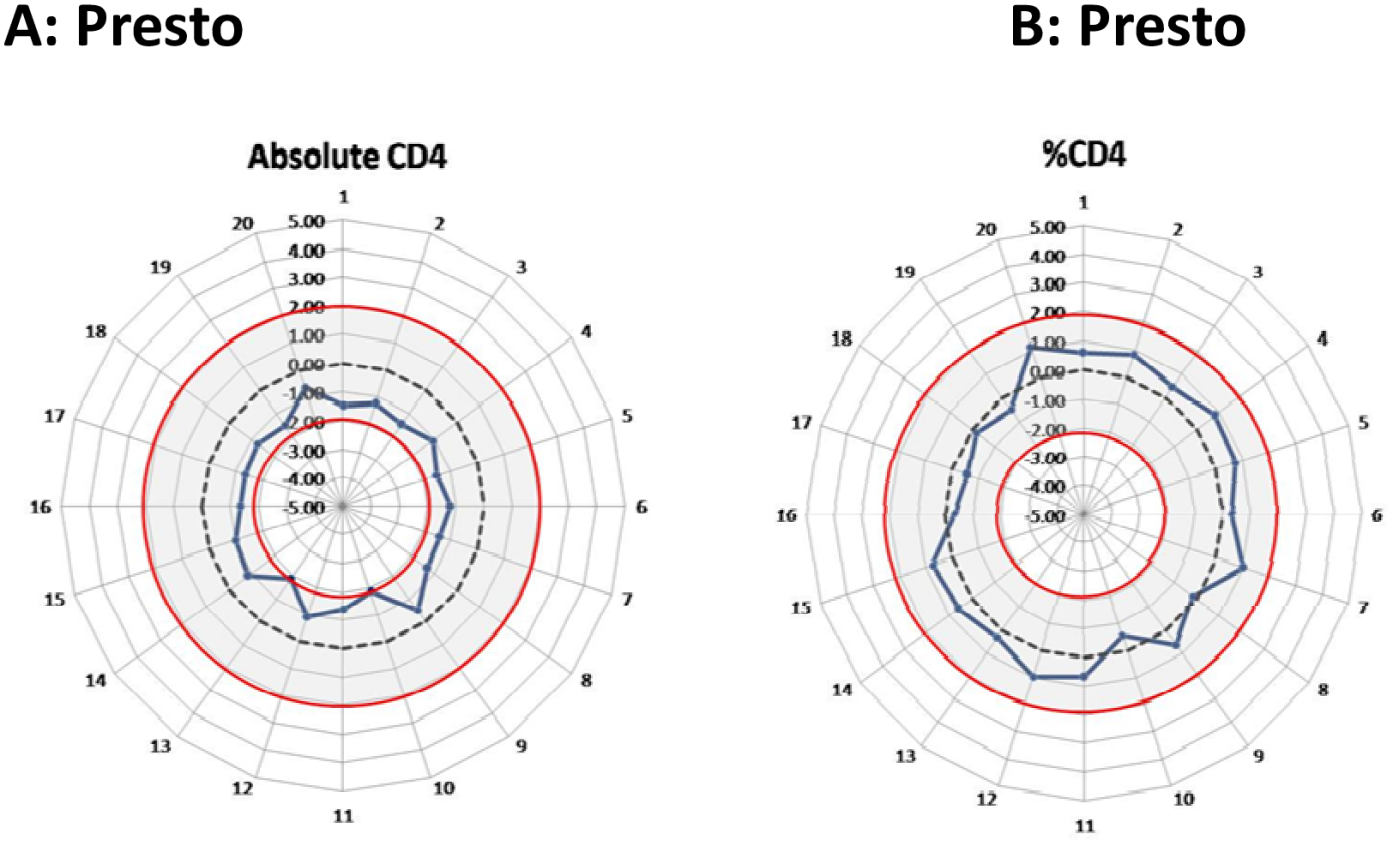
FACSPresto^™^ performance with EQA material. NHLS external quality assessment (EQA) scheme samples (n = 20) was analysed on the FACSPresto^™^ and SDI values (dark solid line) calculated for (a) absolute CD4 counts and (b) CD4% of lymphocytes. C and D indicate the corresponding performance of PLG/CD4 on the MPL system used as the predicate method in this study. The radar graphs depict the optimal target of zero (dashed line) and the acceptable range of -2 to 2 SDI (red circles with grey shaded area).

A panel of 20 retrospective EQA stabilized blood samples (blind testing) were analysed on the FACSPresto^™^ instrument and SDI values calculated and plotted on radar graphs (Fig 3) (S3 Fig). SDI values calculated for both absolute CD4 counts and CD4% of lymphocytes were within the acceptable 2SDI range (-2 to 2) (Fig 3). Comparative results for the predicate method was within acceptable limits (-0.19–1.4 and -0.6 to 1.25 for absolute CD4 and CD4% of lymphocytes respectively). Relative to the trimmed pool consensus mean of 850 participants on the EQA scheme, the Presto instrument produced consistent results lower than the consensus trimmed pool mean across multiple trial samples for absolute CD4 count assessment. CD4% performance on EQA samples (Fig 3B) showed consistent close grouping around the target with SDI values between -1 and 1.

In South Africa, to extend laboratory services into under-serviced areas without access to an existing CD4 service, POC testing has been incorporated, as the Integrated Tiered Service Delivery Model (ITSDM) [50]. The extended laboratory services include two new levels of service, i.e. level 1 as true POC at clinics and level 2 as a mini-laboratory operating multiple POC systems. Although the FACSPresto^™^ instruments are developed to be used for near-patient testing, the impact of sample age on results was assessed in this study for potential use at ITSDM POC level 2 mini-labs where samples could potentially travel from within a 50km radius for testing (>24 hours old). In the eventuality that samples are delayed being referred for testing, an extended 5-day window-of-analysis was performed on five patient samples, with no significant changes in absolute CD4 or CD4% of lymphocyte results (%CV’s <10%), indicating that the system would be able to produce accurate results even on samples that are older than 24 hours.

### Phase II (Clinic testing on capillary samples)

Samples were tested on site at the Witkoppen Health Care Facility using finger stick filled FACSPresto^™^ cartridges from consenting patients, with an average age of 37.4±10.8 years. These results were compared to laboratory EDTA pipetted Presto^™^ cartridges and the PLG/CD4 predicate method, to assess the impact of sampling on result generation and accuracy against predicate method when the system is used under typical clinic conditions (Table 2). Of the 135 samples tested, three did not result either CD4 absolute count of CD4%; with 2 due to internal QC failure (error rate of 1.4%). Absolute CD4 counts as per FACSPresto^™^ in the clinic included a range between 37–1504 cells/μl, with a mean of 560cells/μl. Of the 132 samples, only two had a CD4 count <100cells/μl, 35 had a count of 100–349, 22 had a CD4 count of 350–499 and 73 a count >500cells/μl, supporting the high median and mean CD4 value observed. Of the remaining 132 resulted samples tested at the clinic, results and blood samples for only 118 patients could be retrieved for comparative retesting on the FACSPresto^™^ in the laboratory (capillary vs. venous blood sampling).

**Table 2:** Summary of Phase II validation results.

	A) Predicate PLG/CD4 vs. Clinic FACSPresto (accuracy)		B) Laboratory FACSPresto vs. Clinic FACSPresto (venous vs. capillary sampling)	
	Absolute CD4 count	CD4% lymphocytes	Absolute CD4 count	CD4% Lymphocytes
**Number of samples (n)**	118	118	118	118
**%Similarity Mean ±STDev (Range)**	107.6±10.56	96.7±5.8	105.1±8.8	95.3±5.8
**%Similarity %CV**	9.81	6.08	8.38	6.18
**Bland-Altman bias (Mean ±STDev)**	65.2±99.3	-153±2.46	50.2±92.79	-2.8±2.73
**Bland-Altman 95% LOA**	-129.5 to260.6	-6.36 to 3.29	-131.7–232.1	-9.22 to 2.48

Capillary filled FACSPresto^™^ compared to (A) predicate PLG/CD4 method and (B) venous blood manually filled cartridges in the laboratory (FACSPresto^™^) (S2 Table)

#### Accuracy of clinic results (platform accuracy using capillary sampling)

Clinic collected EDTA venous samples were compared to the predicate method and the laboratory based FACSPresto^™^ (Table 2). A %similarity of 107.6±10.6% and 105.1±8.8% (CV’s 9.8 and 8.4%) was calculated respectively for absolute CD4 count whilst 96.7±5.8 vs. 95.3±5.8 with a corresponding %CV of ˜6% for CD4% of lymphocytes were noted (Table 2A), confirming Phase I results of slight over-estimation of absolute CD4 counts by the FACSPrest^™^ instrument against predicate. The slight under-estimation (<5%) of the CD4% parameter, as per Table 2, was not clinically significant and confirmed the excellent precision to predicate testing noted in Phase I.

#### Comparison of capillary sampling vs. venous sampling on FACSPresto^™^ system

Samples collected through capillary bleeding tested on site using a FACSPresto^™^ were re-tested on the laboratory-based FACSPresto^™^, using venous blood to determine the impact of capillary sampling on accuracy or result reporting (Table 2B). The %similarity for both absolute CD4 count and CD4% of lymphocytes were 105.1±8.8% and 95.3±5.8% with corresponding CV’s of <10%. The bias for absolute CD4 count was 50.2±92.8 (95% LOA of -131.7 to 232) and -2.8±2.7 (95% LOA of -9.2 to 2.5) for CD4% of lymphocytes. Bias data confirmed no significant differences between samples tested at the clinic or laboratory using the FACSPresto^™^ capillary fill vs. FACSPresto^™^ venous fill vs. the reference laboratory.

**Fig 4:**
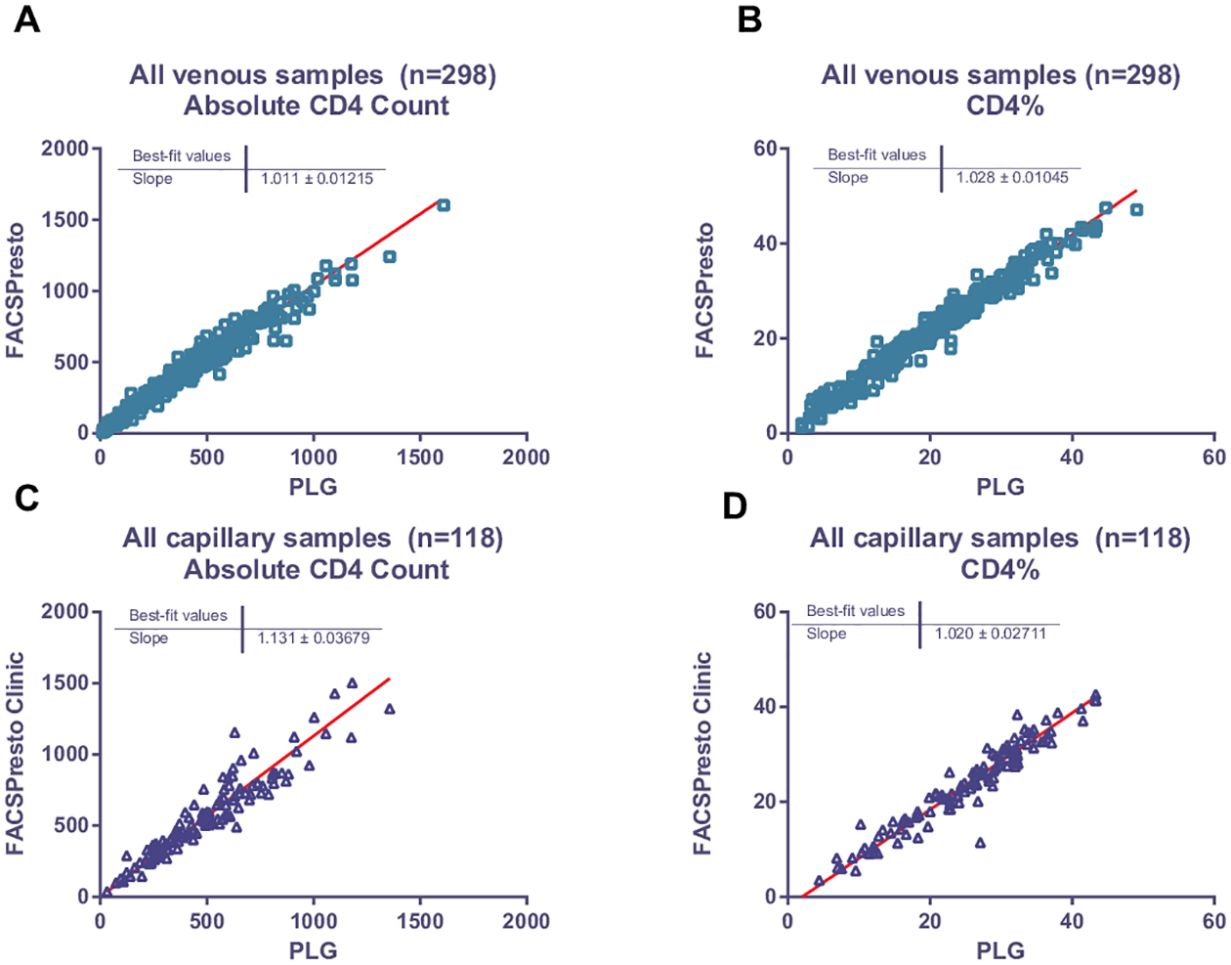
Deming regression analysis. Deming regression analysis was done on venous sampling (A and B) from phase I and II (n = 298) and capillary sampling (n = 118) (C and D) for both absolute CD4 count (A and C) and CD4% of lymphocytes (B and D).

Deming regression analysis of all venous filled samples vs. predicate PLG, and capillary filled samples vs. predicate, confirmed good overall correlation between platforms and sampling methodology (Fig 4) (S4 Fig). Data for Phase I (n = 179) and II (n = 119) using venous blood filled cartridges were combined as there were no significant differences in performance between the two arms of the study using venous sampling.

Sensitivity and specificity analyses of the FACSPresto^™^ against the predicate method, using both venous and capillary blood filled cartridges (Table 3), was reported at both the 350 and 500cells/μl thresholds. This analysis could not be done for capillary sampling at <100cells/μl, as only three samples had a CD4 count <100. As expected, sensitivity at <100 cells/μl for venous sampling was only 70% due to the 10% over-estimating rate of absolute CD4 count values at this level using the FACSPresto^™^ instrument. For thresholds of 350 and 500cells/μl, sensitivity was >80% with specificity of >95%, with overestimation of 8% at these thresholds. Both values were slightly lower for capillary fill at both thresholds (89 vs. 87% and 87 vs. 83% respectively). No significant difference in specificity was noted at the 500cells/μl threshold between sampling methods. False positive rates were <3% at any given threshold or sampling method, with varying false negative rates due to a consistent mean of 6% over-estimation of absolute CD4 counts overall.

**Table 3:** Sensitivity and Specificity analysis.

	<100 cells/μl	<350 cells/μl		<500 cells/μl	
	*FACSPresto Venous*	*FACSPresto Venous*	*FACSPresto Capillary*	*FACSPresto Venous*	*FACSPresto Capillary*
**Sensitivity: (%) (95%CI)**	70.97 (54.99–86.95)	89.92 (84.51–95.33)	79.5 (66.81–92.16)	86.98 (82.22–91.74)	80.95 (71.26–90.65)
**Specificity: (%) (95%CI)**	98.5 (97.04–99.96)	98.8 (97.34–100)	97.5 (94.08–100)	98.13 (95.52–100)	98.21 (94.75–100)
**False Positive Rate: %) (95%CI)**	1.5 (0.04–2.96)	1.17 (-0.4–2.66)	2.5 (-0.9–5.92)	1.88 (-0.7–4.48)	1.79 (-1.68–5.25)
**False Negative Rate: (%) (95%CI)**	29.03 (13.05–34.43)	10.08 (4.67–14.38)	20.51 (7.84–29.1)	13.02 (8.26–18.83)	19.05 (9.35–2.45)
**PPV: (%) (95%CI)**	84.62 (70.75–98.48)	98.16 (95.6–100)	93.94 (85.8–100)	98.82 (97.18–100)	90.7 (84.56–96.84)
**NPV: (%) (95%CI)**	96.96 (94.57–98.82)	93.65 (90.17–97.13)	90.7 (84.56–96.84)	80.62 (73.7–87.4)]	91.6 (86.61–96.58)

Summary of sensitivity and specificity analysis with reference to the positive predictive value (PPV) and negative predictive value (NPV) of venous (EDTA) vs. capillary blood (fingerstick) filled cartridges on the FACSPresto^**™**^ instrument vs. the MPL/CellMek PLG/CD4predicate method at 100 (no data for capillary sampling), 350 and 500cell/μl thresholds.

## Discussion

The Becton Dickinson FACSPresto^™^ near-patient, dedicated point-of-care CD4 instrument was launched recently. This study was conducted to assess the performance of this analyzer, under ideal laboratory conditions using venous blood sampling, as well as how the manufacturer intended the equipment to be used, in a typical health care clinic with capillary blood sampling. Our study revealed reliable CD4 reporting of the Presto^™^ irrespective of whether venous or capillary ‘finger stick’ blood was tested. Although studies with similar cartridge-based systems have reported good correlation under ideal laboratory conditions, in field studies, using capillary sampling, varying accuracy and consistency has been reported [29, 30, 33, 51]. The outcomes for venous blood testing reported here are similar to previous reports where venous sampling was used [27–29, 31–33, 51]. Testing with venous blood revealed a slight but consistent over-estimation of 7% for absolute CD4 count and a 4% over-estimation for the CD4% of lymphocyte parameter. There was an 8- and 5% over-estimation at thresholds of 350 and/or 500 cells/μl respectively, equivalent to a mean absolute but tight difference between the Presto^™^ and predicate system of 40±45 cells/μl for the absolute CD4 count and 1±1.6% for CD4% of lymphocytes. In contrast, however, the data reveals an improved outcome when used in the field with capillary sampling.

Capillary sampling and testing by FACSPresto^™^ compared well with the laboratory predicate method, as well as with the laboratory-based FACSPresto^™^ (on venous blood), again with a slight overestimation of absolute counts, and <5% under-estimation of CD4% of lymphocytes. Importantly, and in contrast to reports from similar systems, there was no significant difference between different operators using capillary sampling. Similar systems, e.g. results reported for PIMA, showed varying degrees of accuracy and reproducibility, mainly due to the methodology of capillary bleed/cartridge fill with field studies outcomes less encouraging, with poorer precision to predicate and a higher bias with wide limits of agreement (variation of reporting) equivalent to -37.9±179.35 and with correlating % similarity CV’s of up to 23% (vs. <8% with venous sampling) [27–29, 51]. These results are confirmed elsewhere; despite small mean Bland Altman bias reported giving the impression of minimal difference, wide limits of agreement have been reported in most studies, confirmed in a large recent meta-analysis [29, 33, 51]. Preliminary results with other cartridge-based systems like Daktari and MBio have also reveal under-estimation of absolute CD4 counts using venous sampling and relatively poor precision to predicate methodology [24, 52].

The improved precision of capillary sampling vs. predicate noted in the field study described here could be related to previous experience of the nursing staff with capillary bleed sampling for other POC tests. Good field results were obtained with PIMA testing in a controlled and monitored clinic setup in an urban setting with experienced staff, emphasizing the importance of ongoing training for end users [29]. The current study underlies the positive impact on testing precision in the hands of operators with previous experience performing POC sampling. Additional field studies of FACSPresto^™^ testing are awaited to confirm the reproducibility noted in the current study.

Results reported in this study using the FACS Presto^™^ also compared well with those generated on other laboratory-based technologies (i.e. BD FACSCount^™^ and BC Tetrachrome T-cell enumeration). As expected, the best correlation was noted between the FACSPresto^™^ and FACSCount^™^ instruments (both from the same manufacturer, refer Fig 1) [53]. Equivalent performance was also noted during testing of pediatric venous blood samples (data not shown). Inter-instrument variability analysis also showed excellent precision between FACSPresto^™^ instruments (CV’s<5%). Performance with EQA material was satisfactory (SDI between -2 to 2), indicating that the testing facilities utilizing these systems can be enrolled onto the South African EQA scheme. Although internal quality control is built in to each cartridge, laboratories may want to add additional QC measures to assure longitudinal monitoring of instrument performance, using stabilized blood products. In this context, the commercially available Immunotrol stabilized blood was used as additional internal quality control measure in this study, due to its availability in South African CD4 testing laboratories. The potential use of other stabilized blood products needs further investigation as these may be more readily available outside South Africa.

Sensitivity and specificity analysis was included in our study for the purpose of comparison to published data on CD4 POC systems. Sensitivity (>80%) and specificity (>95%) analysis of results obtained on the FACSPresto^™^ in relation to the predicate PLG method, showed differences between venous and capillary sampling, at both the 350 and 500 cells/μl threshold levels (Table 3), with capillary sampling faring slightly worse (6–10% difference between venous vs. capillary methodology at individual threshold). Similar outcomes were also reported by Scott et al for meta-analysis of PIMA reported results, with the biggest differences noted in samples with a count <100cells/μl. A decline in specificity was noted at higher CD4 counts for both sampling methods, with a reported sensitivity of >90% at both threshold and comparative specificity of >80% for the 350 cells/μl threshold [33]. Capillary specificity at a threshold of 500 cells/μl was only 73% compared to 81% at 350 cells/μl.

In the current study, misclassification at both thresholds was upward (fewer patients eligible for treatment) using the FACSPresto^™^, similar to the outcomes reported for PointCare Now and opposite to the mostly downward misclassification (over treatment) by PIMA. Over-estimation of results will likely lead to a higher rate of misclassification at the current threshold of 500cells/μl for both venous (13%) and capillary sampling (21%). However, sensitivity analysis should be reviewed with the results of Bland-Altman bias and LOA to assess the true instrument performance as interpretation in isolation of sensitivity and specificity analysis may give a false sense of performance security to end users. The current study showed satisfactory sensitivity and specificity results (similar to reported on elsewhere); the real value of the FACSPresto^™^ system however lies in the precision and reproducibility noted here, important for monitoring patients.

This study emphasizes the need for new systems to be evaluated against predicate methods in use and not necessarily against predicate platforms of the manufacturer only. This work illustrates that the over-estimation of absolute CD4 counts by FACSPresto^™^ noted here, relative to the predicate PLG method, may disappear when tested against other predicate instruments, i.e. FACSCount and may therefore, not be generalizable. Cross platform analysis in this study confirms the latter, as a smaller bias was noted between instruments from the same manufacturer (Becton Dickinson) than was revealed when compared to the predicate methods from another manufacturer (Beckman Coulter). It further underlies that the outcome of any comparative study is affected by the choice of predicate instruments depending on the predicate method used for POC instrument validation. Bearing this in mind, implementation of POC systems may require a once-off adjustment to result reporting (algorithm) to the predicate used, but only if the POC technology is consistent and reproducible. This may not be possible with POC technology that report relatively small mean bias with wide limits of agreement and inconsistency of result reporting [22, 33, 51, 54].

High error rates/invalid test results make some POC CD4 technologies cumbersome for field use [54]. In this study with the FACSPresto^™^, system errors accounted for <2% of samples tested during Phase I and II (failed on-board QC) with ‘no read’ errors comprising 16%, primarily falling into the group of results with CD4 counts <100cells/μl, in Phase I. Although the lower detection limit of the instrument is stated as 50cells/μl, the number of ‘no reads’ was equally distributed for samples with a CD4 count between 1–49 and that 50–99 cells/μl group. CD4 of <100cells/μl is currently included in the national HIV guidelines for early detection of Cryptococcal antigenaemia and as such a critical cut-off for patients entering the HIV treatment programme [8]. The requirement to re-test ‘no read’ samples for absolute CD4 and CD4% reporting is however of concern. It is cumbersome that samples would need to be re-tested on the FACSPresto^™^ or by conventional flow cytometry methods to confirm the CD4 count and eligibility for early CrAg detection. Only 5.6% ‘no reads’ were however noted for samples with CD4 counts >100cells/μl. The manufacturer could improve the ‘no read’ proportion by focusing some attention on further developing the algorithm to alter the current ‘< 50 cells/μl’ cutoff to ‘< than 100 cells/μl’; it is acknowledged that there is no clinical value in providing accurate CD4 counts in the <100cells/μl range. Other reported error rates for similar cartridge based systems ranged from 5–24% [29, 51, 54, 55].

In South Africa, CD4 testing is performed across a network of National Health Laboratory Service (NHLS) laboratories, employing simplified CD4 flow cytometry methods with improved between and within laboratory precision [36, 37, 48]. The systems in use are semi- and fully automated cytometry systems to facilitate the high volumes of tests. Scaling up of international and national HIV programmes created an increased demand for access to CD4 testing [56–58]. South Africa has responded with proposals for implementation of an integrated tiered service model (ITSDM), to extend the national coverage of the current CD4 laboratory testing service footprint [50]. The ‘POC-Hub’ (tier 2), will serve multiple remote clinics and combine all POC testing required for HIV and TB testing (i.e. CD4, VL, TB, basic chemistry, Hb, etc), employ a lower cadre of technical staff, and be implemented into an existing small basic pathology service laboratory. Further extended service is provided with dedicated POC testing sites (tier 1) implemented into clinics located in hard-to reach places, without reasonable access to an existing service laboratory. In countries with good laboratory infrastructure, point-of-care testing may thus be alternatively considered as a means of extending existing services where implementation of traditional laboratory-based systems is not feasible, rather than utilizing POC for same day treatment initiation onto ART [50, 59].

Reliable POC technologies that compare well to existing predicate methods (as reported here with the FACSPresto^™^) are vital to ensure equivalency and quality of services provided in these remote mini-laboratories. In the proposed ITSDM, higher tier labs will take responsibility for POC testing facilities within their geographical area and ensure that quality, equivalent to laboratory testing is translated to the provision of POC tests in the new extended laboratory tiers. Both the PIMA and especially the Presto^™^ technologies evaluated locally, adequately fulfills these needs, if venous blood is used. The advantage of the FACSPresto^™^ in this context is that the technology is equally reliable with capillary sampling. In addition to good precision (low CV’s), the instrument is capable of doing up to 60 samples per 8-hour day, as opposed to just 10–15 sample capacity described for PIMA [29, 51]. The FACSPresto^™^ is thus a feasible and sustainable alternative to introduce extended laboratory services, where daily volumes between 15–50 samples are expected and testing facilities do not have space for larger cytometer based instruments or have adequate/skilled staff to run more sophisticated cytometry systems.

There are currently many described controversies around the use of POC CD4 technologies [20, 21, 60, 61]. Providing CD4 testing at the point-of-care are known to improve linkage to care, with increased numbers of patients noted to initiate ART [12, 14, 16, 17, 62]. Despite this positive outcome, other studies showed that providing CD4 testing at the POC and establishing immediate eligibility for early ART enrolment does not significantly impact on the long-term patient loss to follow up [11, 12]. Recent data suggests that earlier initiation of ART, irrespective of CD4 count in treatment naïve HIV+ patients, improves overall patient outcome rendering use of CD4 technologies at the POC redundant [63, 64]. Further, the negative impact of implementation of POC CD4 testing on health systems and health economics has been well described [13, 60, 61, 65]. Despite these controversies and projected changes in CD4 testing guidelines, a continued role for use of POC CD4 instruments for patient care is likely, perhaps still in some instances to define eligibility for treatment for opportunistic infection or for use where access to laboratory testing is limited.

## Conclusion

This study revealed good precision and a small, consistent positive bias of the BD FACSPresto^™^ to the predicate PLG CD4 method, using both venous and capillary blood. The system can facilitate testing of ~50 samples per day, can adequately process stabilized blood material (Immunotrol) for internal quality control monitoring, as well as EQA material, making it an ideal candidate for use in the field or to extend laboratory services where resources are limited or access to laboratories is poor. Although a positive bias was noted in this study, the outcome should be weighed against the advantage of improved precision that will benefit patient care. Further field studies are awaited.

## Supporting Information

S1 Fig 4 way comparison. (XLSX)

S2 Fig Reproducibility. (XLSX)

S3 Fig EQA. (XLSX)

S4 Fig All data from Deming Regression. (XLSX)

S1 Table Phase I venous sampling. (XLSX)

S2 Table Phase II capillary sampling. (XLSX)
